# Chromoblastomycosis after a leech bite complicated by myiasis: a case report

**DOI:** 10.1186/1471-2334-11-14

**Published:** 2011-01-12

**Authors:** Günther Slesak, Saythong Inthalad, Michel Strobel, Matthias Marschal, Martin JR Hall, Paul N Newton

**Affiliations:** 1 SFE Medical Project, Luang Namtha, Lao PDR; 2 Tropenklinik Paul-Lechler-Krankenhaus, 72076 Tübingen, Germany; 3 Luang Namtha Provincial Hospital, Luang Namtha, Lao PDR; 4 Institut de la Francophonie pour la Médecine Tropicale, Vientiane, Lao PDR; 5 Interfakultäres Institut für Mikrobiologie und Infektionsmedizin, Tübingen, Germany; 6 Department of Entomology, Natural History Museum, Cromwell Road, London, UK; 7 Wellcome Trust-Mahosot Hospital-Oxford Tropical Medicine Research Collaboration, Microbiology Laboratory, Mahosot Hospital, Vientiane, Lao PDR; 8 Centre for Clinical Vaccinology and Tropical Medicine, Churchill Hospital, University of Oxford, Oxford, England, UK

## Abstract

**Background:**

Chromoblastomycosis is a chronic mycotic infection, most common in the tropics and subtropics, following traumatic fungal implantation.

**Case presentation:**

A 72 year-old farmer was admitted to Luang Namtha Provincial Hospital, northern Laos, with a growth on the left lower leg which began 1 week after a forefoot leech bite 10 years previously. He presented with a cauliflower-like mass and plaque-like lesions on his lower leg/foot and cellulitis with a purulent tender swelling of his left heel. Twenty-two *Chrysomya bezziana *larvae were extracted from his heel. PCR of a biopsy of a left lower leg nodule demonstrated *Fonsecaea pedrosoi, monophora*, or *F*. *nubica*. He was successfully treated with long term terbinafin plus itraconazole pulse-therapy and local debridement.

**Conclusions:**

Chromoblastomycosis is reported for the first time from Laos. It carries the danger of bacterial and myiasis superinfection. Leech bites may facilitate infection.

## Background

Chromoblastomycosis is a worldwide chronic infection of the skin and subcutaneous tissue, most commonly found in tropical and subtropical areas. It is mainly caused by the fungal genera *Fonsecaea, Phialophora *and *Cladophialophora *that are saprophytes in soil and plants [1-3]. *Fonsecaea pedrosoi *is the commonest agent found in tropical rain forests [1]. Infection occurs by traumatic cutaneous implantation of fungi [1], for example by skin abrasion from wood or thorns and rarely by an insect or leech bite [4,5]. The lower limbs are most commonly infected and the nodular and/or verrucous plaques can develop centripetal satellite lesions. The most frequent complication is bacterial secondary infection, but malignancies have also been recorded [1,2,6]. Diagnosis can be made by direct microscopic demonstration of pathognomonic brown sclerotic cells (also called fumagoid or muriform cells) in skin scrapings [1-3].

## Case Presentation

**Figure 1:**
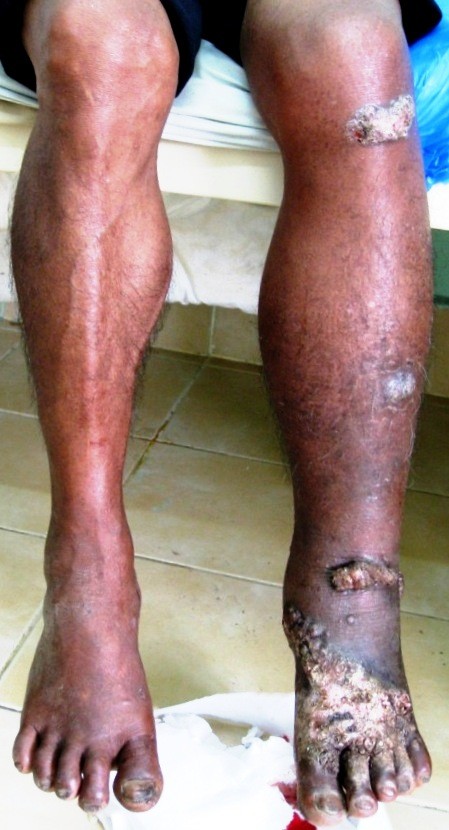
**Lower legs of the patient at presentation with typical lesions on his left foot that spread centripetally up to his knee**.

**Figure 2:**
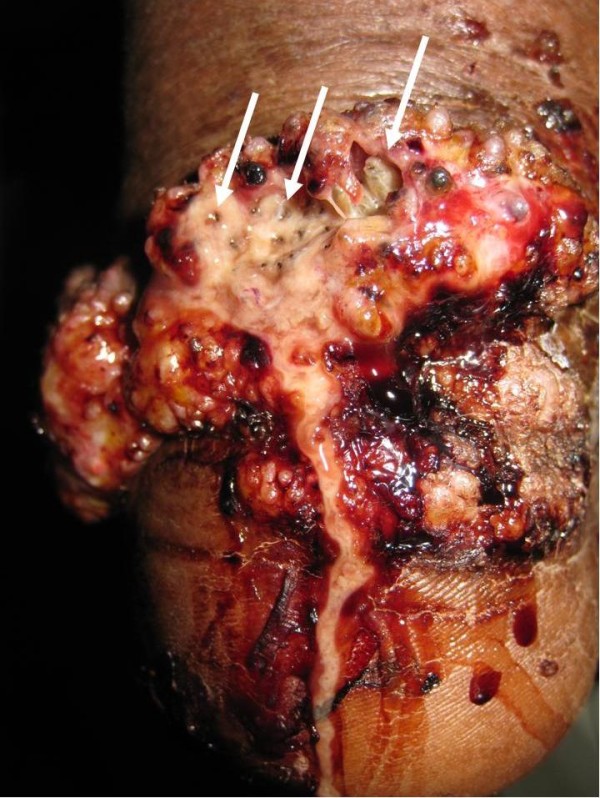
**Patient's heel with groups of deeply burrowed larvae and their black caudal ends**.

An otherwise healthy 72-year-old Khmu farmer was admitted in August 2009 at Luang Namtha Provincial Hospital, northern Lao PDR (Laos), with a painful massive growth on his lower left leg, preventing walking. A red nodule developed one week after a leech bite on the dorsum of the left foot and over ten years, this painless, non-itchy growth spread up to his knee. Three days before admission he developed a painful left ankle with discharge from his heel. On examination he was oriented, afebrile (axillary 37.5°C) with normal vital signs but cauliflower-like masses and several centripetally verrucous oval plaque-like lesions on his left lower leg and foot, with erythema and warmth and a purulent ulcerative very tender swelling of his left heel (Figure 1 and 2).

**Figure 3:**
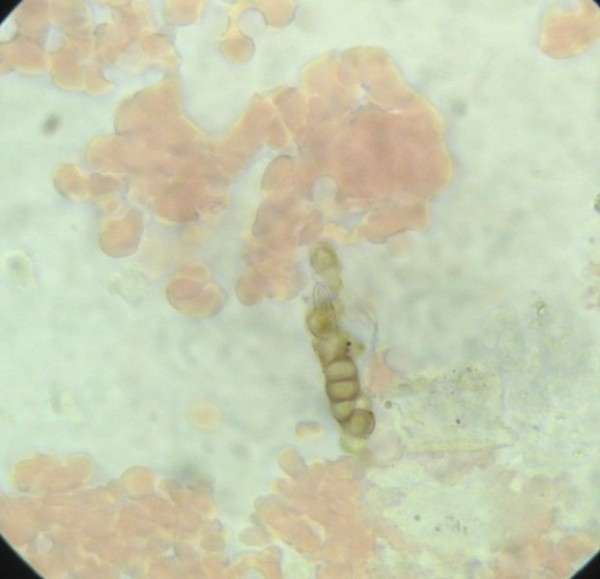
**Characteristic brownish sclerotic cells in skin scrapings (100× objective with oil, wet film)**.

**Figure 4:**
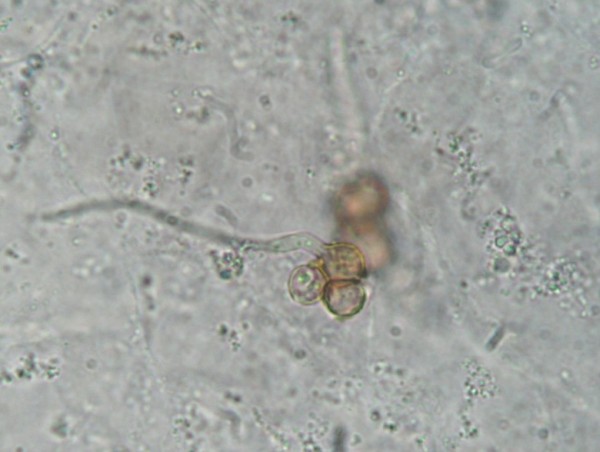
**Sclerotic cells with hyphae (10% potassium hydroxide technique)**.

**Figure 5:**
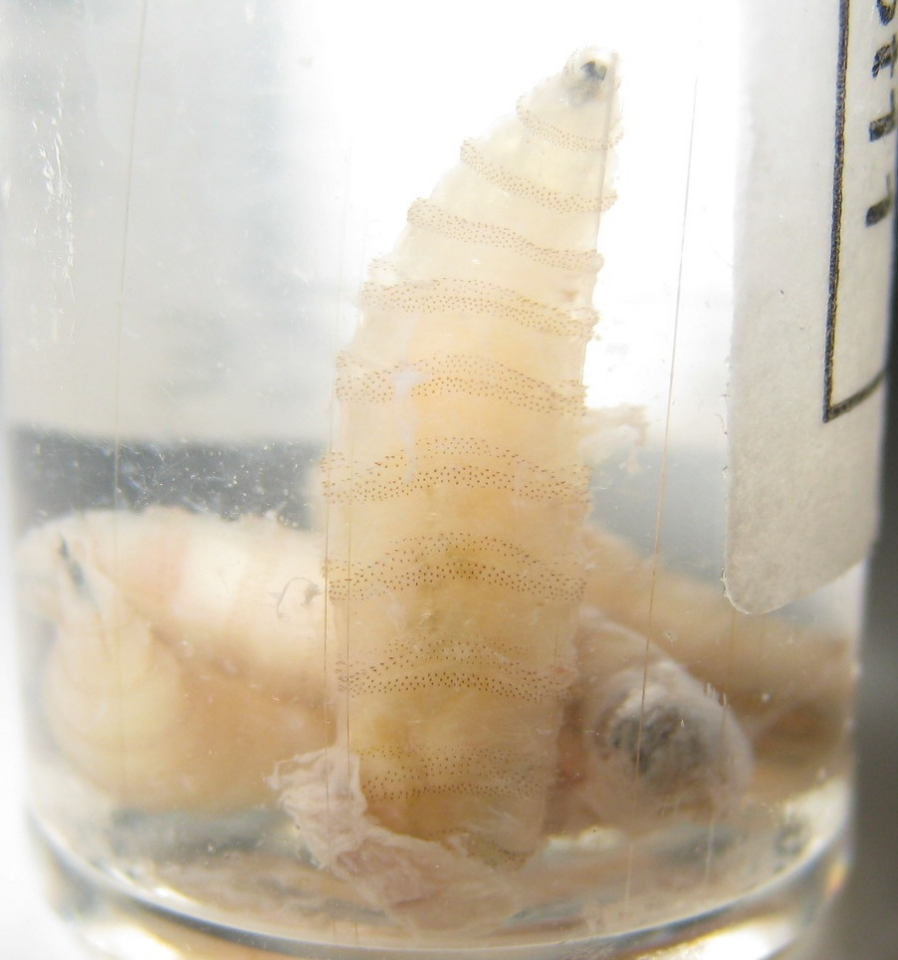
**Third instar larvae of Old World screwworm fly, *Chrysomya bezziana *recovered from the patient's heel with the characteristic bands of black cuticular spines which help to resist extraction**.

He was thought initially to have leprosy or skin cancer, but skin scrapings from the left lower leg lesions revealed typical brownish, round, thick-walled, multiseptate sclerotic cells in a wet film, confirmed with the 10% potassium hydroxide technique [1-3] (Figure 3 and 4). Left lower leg and foot radiographs showed no evidence of bone involvement. He was treated with oral cloxacillin and metronidazole for 1 week, followed by co-trimoxazole, and local iodine-based antiseptics. Bacterial culture of wound discharge grew *Escherichia coli *susceptible to co-trimoxazole by disc diffusion testing (according to CLSI guidelines [7]). During wound dressing on day 3, 22 maggots (fly larvae) were discovered in the heel wound (Figure 5, Additional file 1) and identified as third instar larvae of the Old World screwworm fly, *Chrysomya bezziana *(Diptera: Calliphoridae) [8]. Due to the pathognomonic microscopic findings of sclerotic cells he was diagnosed with chromoblastomycosis and started on itraconazole 400 mg/d monthly pulse therapy [5] on day 18 and a surgical debridement of all skin lesions was performed on day 21.

In order to confirm the diagnosis, and as fungal cultures were not available in Laos, identification by PCR was attempted at Tübingen from heated and ethanol-treated tissue. DNA extraction was performed from each of three tissue samples of about 3 mm in diameter from the patient's lower leg. Fungal DNA was amplified with two different PCR-protocols, using primers of the internal transcribed spacer (ITS) 1 and 4 region [9,10] and the conserved 18S subunit of the rRNA gene [11] with 2.5 μl of DNA-extract applied in each PCR reaction. PCR-products were subsequently sequenced as described [11] and compared with Basic Local Alignment Search Tool (BLAST). PCR using ITS primers remained negative in all 3 samples, whereas 18S rRNA-PCR was positive in one of the 3 samples. Subsequent sequencing revealed 100% similarity with *Fonsecaea pedrosoi, monophora*, and *F*. *nubica*. The sequence was published in GenBank (accession number HQ616145).

**Figure 6:**
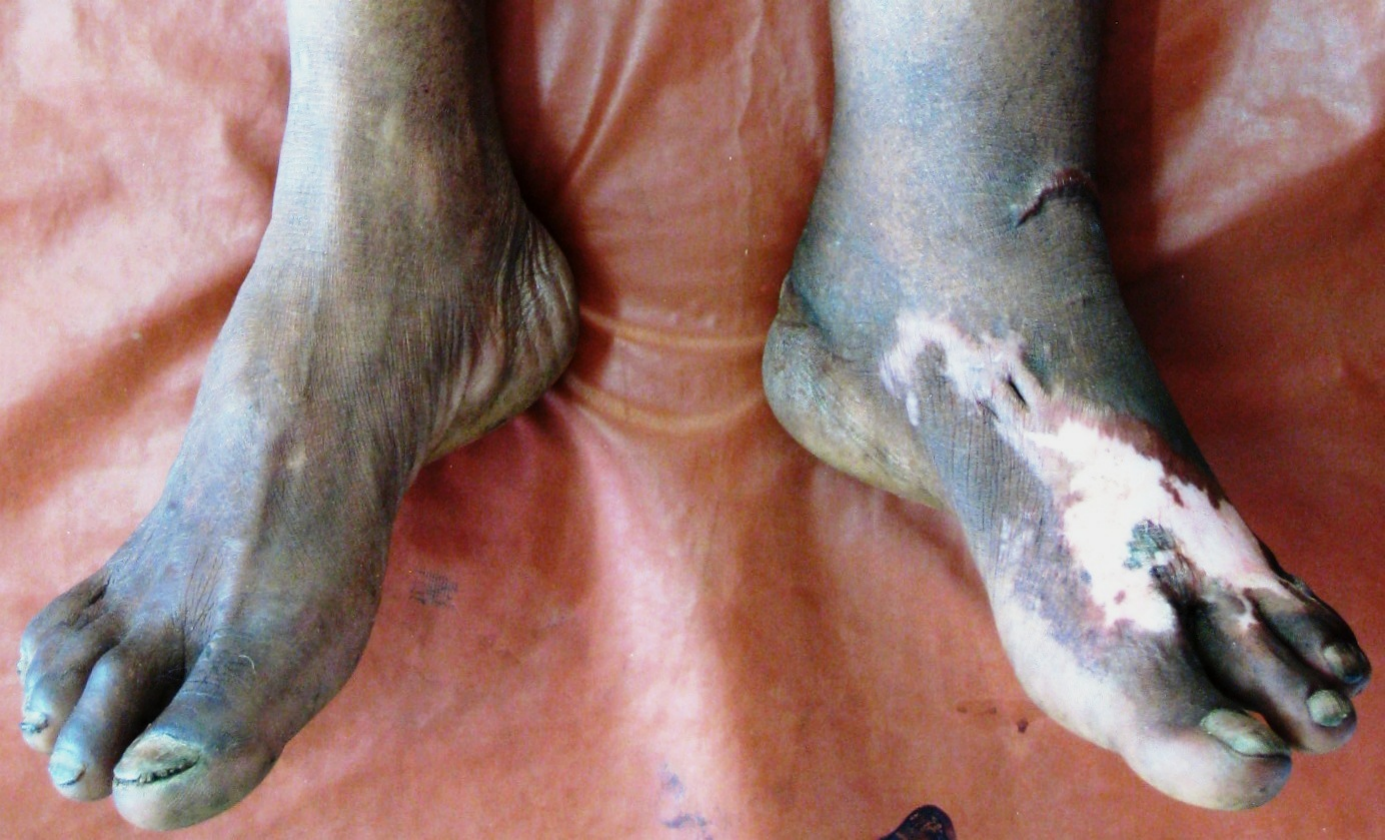
**Healed skin lesions 8 months after initiation of antifungal treatment and surgical debridement**.

After 4 months, oral terbinafin (500 mg/day, later 750 mg/day) for 9 months was added. Local terbinafin ointment was also applied for 6 months. The patient's left lower leg and foot healed without lesions but with some residual swelling (Figure 6).

## Discussion

Chromoblastomycosis has been reported from neighbouring Thailand and China [5,12], and it is likely to be endemic in Laos. Its slow growth and variable appearance may result in confusion with skin cancer, eczema, psoriasis, or leprosy, as in our patient. Direct microscopic identification of muriform/sclerotic cells is diagnostic [1-3] but doctors have to be aware of this differential diagnosis. Treatment remains challenging, especially in financially-poor countries. Antifungals needed to be given for at least 6-12 months, often combined with physical treatments such as surgery, cryotherapy and thermotherapy. Cure rates range from 15% to 80% [1]. Despite being the most common aetiological agent, *F. pedrosoi *appears to be less sensitive to antifungal therapy than either *C. carrionii *or *P. verrucosa *[1,2]. However, terbinafin has shown high tolerability and efficacy especially against *F. pedrosoi*, even in imidazole-refractory cases [13] and dual therapy with itraconazole and terbinafin is recommended [1]. Which species of *Fonsecaea *was responsible remains unclear in our patient. The gene sequence analysis using 18S primers, in contrast to ITS primers, which have been used for *Fonsecaea *species identification in pure cultures [14-16], cannot distinguish between *Fonsecaea pedrosoi*, *F. monophora *and *F*. *nubica *[17]. The finding that only one sample was positive with 18S primers might be due to a very low fungal load or an alteration during the sample processing and transport from remote northern Laos. PCR with 18S primers may have a higher sensitivity for the detection of *Fonsecaea *spp. from biopsy material compared to ITS primers, however, PCR diagnosis of *Fonsecaea *spp. is not yet standardized.

It is possible that the patient acquired the infection via the leech bite or subsequently through the wound. The injection of platelet inhibitors during leech bites [18] might impair the local skin immunity increasing the risk for fungal infections. The coagulation system overlaps with the immune system and many acute-phase proteins of inflammation are also involved in the coagulation process. Platelet microbiocidal proteins and konocidins have shown to exert strong efficacy against bacteria and fungi [19]. To our knowledge this is the second report of chromoblastomycosis associated with a leech bite [5]. Since leeches are typically found in humid tropical areas, as is chromoblastomycosis, there might be an important but overlooked association. Leech bites have been associated with *Aeromonas hydrophilia *infections, complicating 2.4% to 20% of medical leech therapies [20], but might also be involved in the pathogenesis of other tropical soil-related infections, such as *Chromobacterium violaceum *septicaemia [21]. Ingested blood in leeches' digestive tract can contain various pathogens, including HIV and hepatitis B, that, although unproven, could be re-injected into another host by regurgitation during the manipulation of leech removal [22].

Bacterial superinfection is a relatively common complication of chromoblastomycosis [1,2] but myiasis does not appear to have been reported. *Cochliomyia hominivorax, Chrysomya bezziana*, and *Wohlfahrtia magnifica *are the most common flies worldwide causing human wound myiasis. *Chrysomya bezziana *is the most common cause in India and Southeast Asia [23]. Although scientific evidence from Laos is scarce, old clinical descriptions indicate that myiasis has been a common poverty-related complication of neglected wounds in remote areas of Laos [24]. Massive tissue destruction, bone erosion, and death can occur from infestation by Old World screwworm larvae [23]. Our patient had only three of the reported predisposing factors for wound myiasis including advanced age, poor social condition and hygiene, but did not have diabetes, vascular occlusive disease, alcoholism, mental/psychiatric illness, or physical disability [23]. However, the skin lesions of chromoblastomycosis could facilitate fly eggs deposition and might represent another risk factor for human wound myiasis. Although a potentially serious complication, the larval infestation and associated pain had the benefit here of prompting our patient to seek medical attention, which led to the root cause of his ten-year condition being successfully diagnosed and treated.

## Conclusions

Chromoblastomycosis is reported for the first time from Laos and should be considered in the differential diagnosis of chronic skin diseases. Leech bites might facilitate infection and bacterial superinfection and myiasis may occur.

## Consent

Written informed consent was obtained from the patient for publication of this case report including pictures and video.

## Competing interests

The authors declare that they have no competing interests.

## Authors' contributions

GS and SI were the attending physicians who looked after the patient. MS helped with the microscopic diagnosis from skin specimen. MH identified the larvae. GS, SI, and PNN wrote the first draft and all authors revised it. All authors have read and approved the final version.

## Pre-publication history

The pre-publication history for this paper can be accessed here:

http://www.biomedcentral.com/1471-2334/11/14/prepub

## Supplementary Material

Additional file 1 **Videoclip showing perpendicular scraping movements of maggots in the patient's very tender heel that initially were overlooked**. Click here for file
